# A case report—“When less is more”: controlled inpatient reduction of anticholinergic burden in a patient with clozapine-resistant schizophrenia

**DOI:** 10.3389/fpsyt.2023.1222177

**Published:** 2023-07-31

**Authors:** Milica Pjevac, Liam Korošec Hudnik

**Affiliations:** Department for Intensive Psychiatric Treatment, University Psychiatric Clinic Ljubljana, Ljubljana, Slovenia

**Keywords:** anticholinergic burden, treatment-resistant schizophrenia, cognition, clozapine blood level monitoring, delirium

## Abstract

The functional status of an individual with schizophrenia is the defining factor in their quality of life and is closely associated with cognitive abilities, which are impaired in individuals with schizophrenia and considered to be the core symptom of the disorder. The use of psychopharmacotherapy can also have a significant impact on cognitive functioning. The relationship between clozapine treatment and cognitive impairment in individuals with schizophrenia is an intricate one. While some studies have reported a positive effect of clozapine on learning and memory, other studies have found that patients treated with clozapine experienced a decline in cognitive functioning in particular areas. In particular, attention and memory have been shown to deteriorate with rising plasma levels of clozapine. This effect may be attributed to its anticholinergic effect. A reduction in the medication related to anticholinergic burden has been previously found to improve cognitive abilities. In the presented case, we describe a psychotic relapse with delirium symptoms in a patient on clozapine treatment with potentially toxic clozapine blood level. The symptoms of delirium subsided after a clozapine dose adjustment. Gradually lowering the initially very high anticholinergic burden improved the patient's cognitive functioning.

## Introduction

Clozapine is the most efficient medication for treatment-resistant schizophrenia, which occurs in ~30% of schizophrenia patients, 12–20% of whom are clozapine-resistant (1, 2). Despite its superior efficacy and antipsychotic, anti-aggressive, anti-suicidal, and anti-impulsive properties, it is reserved for resistant cases due to its potentially severe side effects (3). Close monitoring for agranulocytosis, myocarditis, and intestinal obstruction is necessary. Only 40% of resistant schizophrenia patients, however, will fully respond to clozapine, and of these, between 40 and 70% will develop clozapine resistance over time (4). Other side effects, such as weight gain, hypersalivation, sedation, tachycardia, and seizures, can complicate treatment and require additional medications (5).

Cognitive impairment is among the core features of schizophrenia, and clozapine's prominent anticholinergic properties may further compromise cognitive function. Joshi et al. showed that patients with schizophrenia with higher cumulative ACB (Anticholinergic Cognitive Burden Scale) scores had significantly greater cognitive impairment, with optimistic preliminary results after lowering the anticholinergic burden (6). The ACB scale assigns a dose-independent rating to each medication based on its anticholinergic properties. This is not an exact or accurately quantifiable measure of anticholinergic burden, but rather an estimate that takes into account the known characteristics and data of each given medication. When anticholinergic side effects are present or best avoided, such as in geriatric patients, or are a clinically evident problem, the scale is useful in determining which medications contribute significantly to the anticholinergic burden and can support clinicians in making an informed decision to optimize the prescribed medication regimen. Additionally, delirium has been described as an uncommon side effect of clozapine treatment, probably due to its central anticholinergic properties (7). Clozapine's low antidopaminergic activity in combination with simultaneous acetylcholine decreases may also contribute to the development of delirium, especially hyperactive forms (8).

In the case presented, we describe a real-life clinical example of treatment-resistant schizophrenia in which symptoms of the underlying psychotic disorder overlap with adverse effects of clozapine and other prescribed medications. This case is an example of complex polypharmacy. The objective is to elucidate the clinical importance and complexity associated with distinguishing between primary symptoms and potential adverse effects of medications. Our main emphasis lies on anticholinergic side effects, particularly the cognitive impairment resulting from the anticholinergic burden of medications. These cognitive impairments may coincide with the inherent cognitive deficits observed in schizophrenia. Furthermore, in the case presented, anticholinergic delirium is shown to overlap with acute exacerbations of positive symptoms. This represents an important issue, as polypharmacy is extremely common in the treatment of schizophrenia. Our aim is to emphasize the significance of identifying and distinguishing between the primary symptoms and medication-related adverse effects and to demonstrate the benefits, in this case in terms of cognitive and functional status, of adjusting the medication accordingly. Additionally, we would like to stress the benefits of an interdisciplinary approach in cases of complex polypharmacy, in particular consultation with a clinical pharmacist, as this has previously been shown to improve clinical outcomes (9).

## Case description

A 57-year-old female patient with treatment-resistant and arguably clozapine-resistant schizophrenia on clozapine treatment presented with acute psychotic symptoms. She exhibited bizarre delusions, auditory and possibly tactile and visual hallucinations, and disorganized speech, and there were reports of aggressive tendencies. The patient had been treated for paranoid schizophrenia since 1995. During this period, she had been hospitalized several times in our clinic due to acute exacerbations of psychotic symptoms. In 2016, with her consent, she was admitted to a specialized long-term care facility because of an impairment in social functioning and a severely diminished capacity for independent functioning.

Initially, the subjects was prescribed the same medications and dosages as in the long-term care facility, i.e., clozapine 700 mg/day divided into three daily doses, zuclopenthixol decanoate 200 mg LAI every 2 weeks, zuclopenthixol 25 mg/day (oral), biperiden 6 mg/day, flurazepam 15 mg at bedtime, and lorazepam 5.5 mg/day. This placed the patient at high risk for confusion, falls, or death due to the high anticholinergic burden on the ACB scale. There were no reports of recent changes in any of the medications.

During the first week on the ward, she exhibited marked variability in observed clinical presentations, with persistent reports of auditory hallucinations and bizarre delusions as well as superimposed reports of episodes of extremely disorganized behavior with escalations of psychomotor agitation, apparent disorientation, and confusion. These episodes usually occurred at night and were related to severe insomnia, which led to the use of additional benzodiazepines or haloperidol. The nursing staff regularly reported that she seemed to be experiencing visual hallucinations during these periods.

We hypothesized that the patient was experiencing episodes of delirium, possibly anticholinergic delirium. A general workup, including a clinical examination, laboratory tests, and microbiologic assays, was performed to exclude other possible precipitating factors or causes of delirium. A plasma clozapine level was obtained during Week 2 of hospitalization; the results were 1,130 μg/L, markedly above the current consensus for the minimum plasma level required to achieve a clinical response (350 μg/L) and also above the recommended laboratory alert level of 1,000 μg/L (10). The patient, however, did not present with other possible symptoms of clozapine toxicity (seizures, hypersalivation, tachycardia, hypotension, sedation, coma, and respiratory depression). Possible partial epileptic seizures were excluded by the EEG. We consulted an in-hospital clinical pharmacist and proceeded to gradually reduce the clozapine dose by ~25 mg per week, reaching 450 mg/day by Week 11. This resulted in a plasma clozapine level of 435 μg/L at Week 15 of hospitalization. The clozapine/N-desmethylclozapine ratio also shifted from 1.82 to 0.98 (both levels obtained at steady-state conditions). The relatively high doses of benzodiazepines prescribed could, of course, further worsen the delirium, so we gradually reduced the dose but decided against abrupt discontinuation to avoid severe withdrawal symptoms, given that the patient had been on these medications for a long time leading up to this hospitalization. The signs of delirium slowly dissipated. The sleep-wake cycle was re-established, and there was no need for flurazepam or additional hypnotic medication at bedtime.

To further reduce the anticholinergic burden, biperiden was gradually withdrawn, and the interval between doses of zuclopenthixol 200 mg LAI was increased to every 4 weeks. To avoid possible rebound symptoms, the anticholinergics were gradually tapered, and we regularly screened for a possible worsening of psychotic features. In the case of biperiden, no information was provided by the long-term care facility as to why this medication was prescribed in the first place, but no signs of movement disorders were recorded following its discontinuation. Additionally, to ensure sufficient control of the symptoms of the primary disorder, clozapine therapy was augmented with the addition of amisulpride, 1,000 mg/day by Week 21, and lamotrigine, 200 mg/day by Week 17. Her Mini-Mental State Examination scores improved from 13/30 on Week 10 to 22/30 on Week 21.

**Figure 1:**
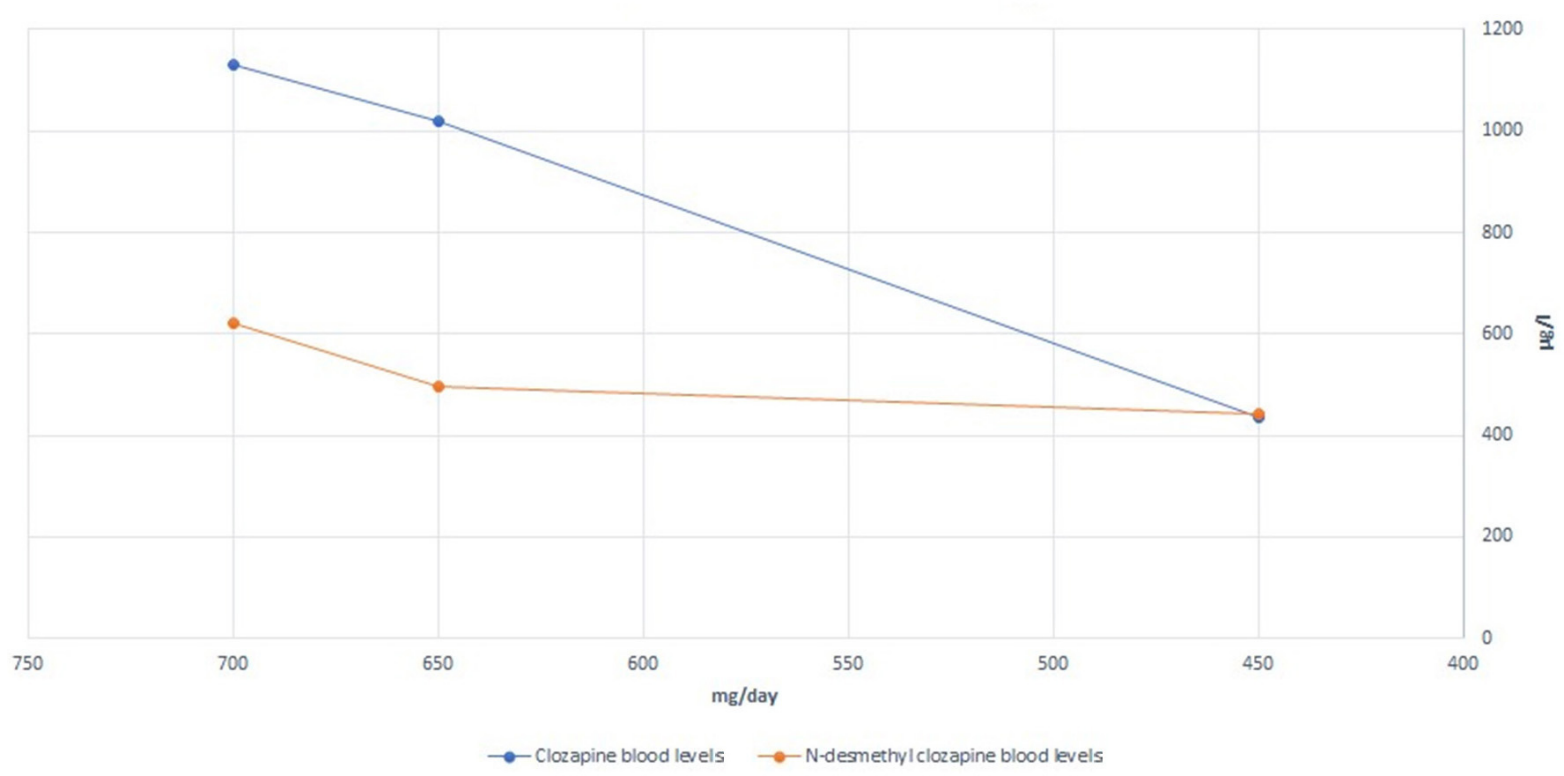
Clozapine blood level monitoring.

In total, the patient was hospitalized for 22 weeks. She was discharged from the hospital with the following medications: clozapine 450 mg/day, amisulpride 1,000 mg/day, lamotrigine 200 mg/day, zuclopenthixol 200 mg LAI every 4 weeks, zuclopenthixol 30 mg/day, divided into three daily doses, and lorazepam 6.5 mg/day, divided into three daily doses, and with the option of a gradual dose reduction in the long-term care facility. In an attempt to improve her cognitive function even further, an acetylcholinesterase inhibitor was empirically added to her medications prior to discharge. She was prescribed a transdermal patch with 9.5 mg of rivastigmine/day. A detailed presentation of the changes in medications and recorded clozapine and N-desmethylclozapine levels is shown in Figure 1 and Table 1.

**Table 1:** Timeline.

		**Treatment**	**Problems**
Week 1–4	November	Zuklopentixol 25 mg/day • Biperiden 6 mg/day • Fluzepam 15 mg/day • Lorazepam 5.5 mg/day • Clozapine 700–650 mg/day • Zuklopenthixol long-acting 200 mg/2 weeks	22.11. Brief psychiatric rating scale (BPRS) = 57 • Delirium observation scale (DOS) = 52 • 7.11. Therapeutic drug monitoring (TDM): 1,130 μg/l • 28.11. TDM: 1,020 μg/l • Psychosis, delirium
Week 5–8	December	Zuklopentixol 30 mg/day • Biperiden 3 × 2 mg-ex at week 8 • Lorazepam 4 mg/day • Flurazepam 15 mg/day • Clozapine 525 mg/day	29.12. Anticholinergic burden score (ACB): 7 • Mini-mental state examination (MMSE): 13/30 • Psychosis
Week 9–12	January	Clozapine 450–400 mg/day • Zuklopentixol 50 mg/day • Lorazepam 7.5 mg/day • Fluzepam 15 mg/day • Lamotrigine 25–100 mg/day • Zuklopentixole LAI 200 mg/4 weeks	30.1. ACB: 5 • MMSE: 13/30 • Psychosis • Cognitive impairment
Week 13–16	February	Clozapine 450 mg/day • Zuklopenthixole 30 mg/day • Lorazepam 7.5 mg/day • Lamotrigine 150–200 mg/day • Amislupride 600 mg/day • Zuklopentixole LAI 200 mg/4 weeks	
Week 17–22	March–April	Zuklopentixole 20 mg/day • Clozapine 450 mg/day • Lamotrigine 200 mg/day • Amisulpride 1,000 mg/day • Lorazepam 6.5 mg/day • Rivastigmine patch 9.5 mg/day • Zuklopentixole LAI 200 mg/4 weeks	15.3.2023- MMSE: 22/30 • 22.3.: ACB 4

## Discussion

The functional status of an individual with schizophrenia is the defining factor in their quality of life and is closely associated with cognitive abilities, which are impaired in people with schizophrenia and are considered to be the core symptom of the disorder. The use of psychopharmacotherapy can also have a significant impact on cognitive function.

The relationship between clozapine treatment and cognitive impairment in people with schizophrenia is an intricate one. While some studies have reported a positive effect of clozapine on learning and memory, other studies have found that patients treated with clozapine experienced a decline in cognitive function in specific areas. In particular, attention and memory have been shown to deteriorate with increased plasma levels of clozapine (11, 12). This effect may be due to its anticholinergic activity. A reduction in the medication related to anticholinergic burden has previously been found to improve cognitive function (13).

In this case, the initial plasma clozapine levels were distinctly above the reported plasma levels, beyond which no additional antipsychotic benefit is to be expected in most individuals, while dose-dependent adverse effects become increasingly likely (10, 14). Given both the high anticholinergic burden due to the clozapine plasma levels and other anticholinergic medications, an expected cognitive deficit was objectively shown using the mini-mental state examination. We would also like to point out the possible effect of the change in the clozapine/N-desmethylclozapine ratio between the first and second plasma levels obtained. It has previously been suggested that the ratio of clozapine to N-desmethylclozapine may independently influence cognitive performance due to the different muscarinic agonist/antagonist action profiles between clozapine and N-desmethylclozapine (15). In the present case, the clozapine/N-desmethylclozapine ratio changed to a theoretically favorable lower value. The major enzymes involved in clozapine conversion to N-desmethylclozapine are CYP1A2 and CYP3A4 (16). In the absence of any additional exposure to an enzyme inducer (e.g., smoking, carbamazepine, omeprazole, or phenytoin), this change after a gradual clozapine dose reduction suggests that the N-desmethylation process was previously saturated. Additionally, when administered together, clozapine and zuclopenthixol could potentially compete for the same metabolizing enzymes, specifically CYP3A4 (17), potentially increasing the levels of one or both drugs in the absence of an enzyme inhibitor.

The substantial improvement in cognitive function after clozapine dose reduction and anticholinergic burden reduction suggests cognitive dysfunction to be at least partially secondary to medication side effects and not exclusively a feature of the primary disorder. In addition, it is important to note that benzodiazepines can also markedly contribute to cognitive impairment, so further gradual reduction of the benzodiazepine may lead to additional improvements in her cognitive status.

In clozapine-resistant schizophrenia, antipsychotic polypharmacy is a common practice. Since prominent auditory hallucinations and delusions in the patient persisted despite clozapine plasma levels being above the estimated response threshold on admission (14), clozapine therapy had been augmented with zuclopenthixol, adding to the cumulative anticholinergic burden. Even though the literature does not favor any particular antipsychotic drug as an augmentation to clozapine therapy in cases of inadequate response or residual psychotic symptoms, from a pharmacodynamic perspective, the addition of amisulpride may be beneficial if supplementary dopamine receptor blockade is desired without increasing the anticholinergic burden. Additionally, the reduction in clozapine dose along with the addition of amisulpride has previously been shown to significantly reduce adverse effects compared to monotherapy (18).

Furthermore, clozapine's potent central anticholinergic activity is a risk factor for the development of delirium. As described in the present case, this complication of clozapine treatment, or arguably clozapine toxicity, given the very high plasma levels, led to further worsening of the patient's cognition and functional status. The incidence of delirium in patients treated with clozapine is estimated to be ~5% (19) but may be underrecognized in clinical practice due to the difficulty in identifying and differentiating signs of delirium in psychotic patients. A misdiagnosis of delirium as an exacerbation of the underlying psychotic disorder may lead to a counterproductive and potentially dangerous escalation of the drug dose that precipitated the state of delirium.

## Conclusions

Antipsychotic polypharmacy is extremely common in the management of treatment-resistant schizophrenia. As is evident in the present case, adverse effects of medications can overlap with the primary symptoms of the underlying disorder, and differentiating between the two can present an important clinical challenge and should influence treatment decisions. The anticholinergic burden can severely impair the cognitive and functional abilities of individuals with schizophrenia, but the clinical case presented demonstrates that a gradual and controlled reduction of the anticholinergic burden, with screening for potential worsening of psychotic symptoms, may be an approach to consider in improving functional outcomes. Further research and real-life clinical data are of course needed to support any conclusions, as this case represents only anecdotal data and is an example of extensive polypharmacy, which represents an important limitation for generalization.

## Data availability statement

The raw data supporting the conclusions of this article will be made available by the authors, without undue reservation.

## Ethics statement

Ethical review and approval was not required for the study on human participants in accordance with the local legislation and institutional requirements. Written informed consent for participation was not required for this study in accordance with the national legislation and the institutional requirements. Written informed consent was obtained from the individual(s) for the publication of any potentially identifiable images or data included in this article. Written informed consent was obtained from the participant/patient(s) for the publication of this case report.

## Author contributions

All authors listed have made a substantial, direct, and intellectual contribution to the work and approved it for publication.

## References

[1] Taipale H Tanskanen A Mehtälä J Vattulainen P Correll CU Tiihonen J. 20-year follow-up study of physical morbidity and mortality in relationship to antipsychotic treatment in a nationwide cohort of 62,250 patients with schizophrenia (FIN20). World Psychiatry. (2020) 19:61–8. 10.1002/wps.20699 31922669 PMC6953552

[2] Siskind D Siskind V Kisely S. Clozapine response rates among people with treatment-resistant schizophrenia: data from a systematic review and meta-analysis. Can J Psychiatry. (2017) 62:772–7. 10.1177/0706743717718167 28655284 PMC5697625

[3] Khokhar JY Henricks AM Sullivan EDK Green AI. Unique effects of clozapine: a pharmacological perspective. In: Advances in Pharmacology. Elsevier (2018). p. 137–62. Available from: https://linkinghub.elsevier.com/retrieve/pii/S1054358917300881 (accessed April 27, 2023). 10.1016/bs.apha.2017.09.009 PMC7197512 29413518

[4] de Leon J Schoretsanitis G Smith RL Molden E Solismaa A Seppälä N. An international adult guideline for making clozapine titration safer by using six ancestry-based personalized dosing titrations, CRP, and clozapine levels. Pharmacopsychiatry. (2022) 55:73–86. 10.1055/a-1625-6388 34911124

[5] Miller DD. Review and management of clozapine side effects. J Clin Psychiatry. (2000) 61(Suppl. 8):14–7; discussion 18–19. 10811238

[6] Joshi YB Thomas ML Braff DL Green MF Gur RC Gur RE. Anticholinergic medication burden–Associated cognitive impairment in schizophrenia. Am J Psychiatry. (2021) 178:838–47. 10.1176/appi.ajp.2020.20081212 33985348 PMC8440496

[7] Das A Minner R Krain L Spollen J. Delirium on clozapine: a tale of friend turned foe-A case report. Int J Psychiatry Med. (2021) 56:446–58. 10.1177/0091217420972827 33148081

[8] Hshieh TT Fong TG Marcantonio ER Inouye SK. Cholinergic deficiency hypothesis in delirium: a synthesis of current evidence. J Gerontol A Biol Sci Med Sci. (2008) 63:764–72. 10.1093/gerona/63.7.764 18693233 PMC2917793

[9] Stuhec M Tement V. Positive evidence for clinical pharmacist interventions during interdisciplinary rounding at a psychiatric hospital. Sci Rep. (2021) 11:13641. 10.1038/s41598-021-92909-2 34211019 PMC8249606

[10] Schoretsanitis G Kane JM Correll CU Marder SR Citrome L Newcomer JW. Blood levels to optimize antipsychotic treatment in clinical practice: A joint consensus statement of the american society of clinical psychopharmacology and the therapeutic drug monitoring task force of the arbeitsgemeinschaft für neuropsychopharmakologie und pharmakopsychiatrie. J Clin Psychiatry. (2020) 81:5–8. 10.4088/JCP.19cs13169 32433836

[11] Nielsen J Damkier P Lublin H Taylor D. Optimizing clozapine treatment: optimizing clozapine treatment. Acta Psychiatr Scand. (2011) 123:411–22. 10.1111/j.1600-0447.2011.01710.x 21534935

[12] Savulich G Mezquida G Atkinson S Bernardo M Fernandez-Egea E. A case study of clozapine and cognition: friend or foe? J Clin Psychopharmacol. (2018) 38:152–3. 10.1097/JCP.0000000000000847 29394180

[13] McGurk S. Antipsychotic and anticholinergic effects on two types of spatial memory in schizophrenia. Schizophr Res. (2004) 68:225–33. 10.1016/S0920-9964(03)00123-3 15099605

[14] Meyer JM, Stahl, SM,. The Clinical Use of Antipsychotic Plasma Levels: Stahl's Handbooks. 1st ed. Cambridge University Press (2021). Available from: https://www.cambridge.org/core/product/identifier/9781009002103/type/book (accessed May 8, 2023).

[15] Dal Santo F Jarratt-Barnham I González-Blanco L García-Portilla MP Bobes J Fernández-Egea E. Longitudinal effects of clozapine concentration and clozapine to N-desmethylclozapine ratio on cognition: a mediation model. Eur Neuropsychopharmacol. (2020) 33:158–63. 10.1016/j.euroneuro.2020.01.016 32057590

[16] Pirmohamed M Williams D Madden S Templeton E Park BK. Metabolism and bioactivation of clozapine by human liver in vitro. J Pharmacol Exp Ther. (1995) 272:984–90. 7891353

[17] Davies SJC Westin AA Castberg I Lewis G Lennard MS Taylor S. Characterisation of zuclopenthixol metabolism by in vitro and therapeutic drug monitoring studies: Zuclopenthixol metabolism and interactions. Acta Psychiatr Scand. (2010) 122:444–53. 10.1111/j.1600-0447.2010.01619.x 20946203

[18] Zink M Knopf U Henn FA Thome J. Combination of clozapine and amisulpride in treatment-resistant schizophrenia - case reports and review of the literature. Pharmacopsychiatry. (2004) 37:26–31. 10.1055/s-2004-815471 14750045

[19] Centorrino F Albert MJ Drago-Ferrante G Koukopoulos AE Berry JM Baldessarini RJ. Delirium during clozapine treatment: incidence and associated risk factors. Pharmacopsychiatry. (2003) 36:156–60. 10.1055/s-2003-41201 12905102

